# Mrs. Catherine Cumming

**Published:** 1853-07-01

**Authors:** 


### MRS. CATHERINE CUMMING.

This lady died on Tuesday, the 21st of June, at her own private residence in St. John's Wood. Our readers will remember that Lord St. Leonards, when Lord Chancellor, after having a personal interview with Mrs. Cumming, and hearing a learned and elaborate argument in reference to the question, whether the petition for a traverse should be granted, decided, that Mrs. Cumming was entitled to a new trial. It was generally expected that the question of this lady's insanity would be argued before the Court of Queen's Bench this term, and then left to the decision of another jury; but her lamented death puts, of course, an end to these legal proceedings. For the purpose of obtaining additional medical evidence in support of the allegation of insanity, several medical gentlemen were deputed by the solicitor representing the portion of Mrs. Cumming's family prosecuting the inquiry, to visit and examine Mrs. Cumming. These examinations, (although conducted by the professional gentlemen in a legitimate and kind spirit,) produced a very prejudicial effect upon Mrs. Cumming's bodily health, and induced a train of alarmingly depressing symptoms. It was consequently deemed necessary at once to prohibit a continuance of these irritating investigations, and Dr. Winslow, acting under the authority invested in him by an order of the Court of Chancery, refused to allow any person to see Mrs. Cumming who proposed to visit her for the purpose of testing her mental capacity. An attempt was subsequently made on the part of the solicitor, acting for Mrs. Cumming's family, to induce the court to instruct Dr. Winslow to withdraw his prohibition, but the Lords Justices refused to interfere. The result establishes that they took, not only a just, but a *humane* view of Mrs. Cumming's state. Had this case proceeded to trial, some of the most learned and eloquent men at the bar would have been ranged on both sides, and the battle would have been gallantly fought. We never felt for a moment uneasy as to the final result. Mrs. Cumming died, as she has lived for many years, an *eccentric* but a *sane* woman.

